# Long-term scar quality after hydrosurgical versus conventional debridement of deep dermal burns (HyCon trial): study protocol for a randomized controlled trial

**DOI:** 10.1186/s13063-018-2599-2

**Published:** 2018-04-19

**Authors:** Catherine M. Legemate, Harold Goei, Esther Middelkoop, Irma M. M. H. Oen, Tim H. J. Nijhuis, Kelly A. A. Kwa, Paul P. M. van Zuijlen, Gerard I. J. M. Beerthuizen, Marianne K. Nieuwenhuis, Margriet E. van Baar, Cornelis H. van der Vlies

**Affiliations:** 1 Association of Dutch Burn Centers, Maasstad Hospital, Rotterdam, the Netherlands; 2 Department of Plastic, Reconstructive and Hand Surgery, Amsterdam Movement Sciences, VU University Medical Center, Amsterdam, the Netherlands; 3 Association of Dutch Burn Centers, Burn Centre, Red Cross Hospital, Beverwijk, the Netherlands; 4 Burn Center, Maasstad Hospital, Rotterdam, the Netherlands; 5 Department of Plastic, Reconstructive and Hand Surgery, Erasmus Medical Center, Rotterdam, the Netherlands; 6 Burn Center, Red Cross Hospital, Beverwijk, the Netherlands; 7 Department of Plastic, Reconstructive and Hand Surgery, Red Cross Hospital, Beverwijk, the Netherlands; 8 Department of Surgery, Leiden University Medical Center, Leiden, the Netherlands; 9 Burn Center, Martini Hospital, Groningen, the Netherlands; 10 Association of Dutch Burn Centers, Martini Hospital, Groningen, the Netherlands; 11 Department of Surgery, Maasstad Hospital, Rotterdam, the Netherlands; 12 Trauma Research Unit Department of Surgery, Erasmus MC, University Medical Center, Rotterdam, the Netherlands

**Keywords:** Conventional debridement, Versajet, Hydrosurgery, Tangential excision, Burns, Scar quality

## Abstract

**Background:**

Deep dermal burns require tangential excision of non-viable tissue and skin grafting to improve wound healing and burn-scar quality. Tangential excision is conventionally performed with a knife, but during the last decade hydrosurgery has become popular as a new tool for tangential excision. Hydrosurgery is generally thought to be a more precise and controlled manner of burn debridement leading to preservation of viable tissue and, therefore, better scar quality. Although scar quality is considered to be one of the most important outcomes in burn surgery today, no randomized controlled study has compared the effect of these two common treatment modalities with scar quality as a primary outcome. The aim of this study is, therefore, to compare long-term scar quality after hydrosurgical versus conventional tangential excision in deep dermal burns.

**Methods/design:**

A multicenter, randomized, intra-patient, controlled trial will be conducted in the Dutch burn centers of Rotterdam, Beverwijk, and Groningen. All patients with deep dermal burns that require excision and grafting are eligible. Exclusion criteria are: a burn wound < 50 cm^2^, total body surface area (TBSA) burned > 30%, full-thickness burns, chemical or electrical burns, infected wounds (clinical symptoms in combination with positive wound swabs), insufficient knowledge of the Dutch or English language, patients that are unlikely to comply with requirements of the study protocol and follow-up, and patients who are (temporarily) incompetent because of sedation and/or intubation. A total of 137 patients will be included. Comparable wound areas A and B will be appointed, randomized and either excised conventionally with a knife or with the hydrosurgery system. The primary outcome is scar quality measured by the observer score of the Patient and Observer Scar Assessment Scale (POSAS); a subjective scar-assessment instrument, consisting of two separate six-item scales (observer and patient) that are both scored on a 10-point rating scale.

**Discussion:**

This study will contribute to the optimal surgical treatment of patients with deep dermal burn wounds.

**Trial registration:**

Dutch Trial Register, NTR6232. Registered on 23 January 2017.

**Electronic supplementary material:**

The online version of this article (10.1186/s13063-018-2599-2) contains supplementary material, which is available to authorized users.

## Background

Surgical debridement is an important step in the treatment of patients with deep dermal burns. The purpose is to remove necrotic and infectious materials and to prepare tissue for skin grafting and definitive wound closure [1]. Conventional surgical debridement of acute burn wounds consists of sharp tangential excision of non-viable tissue with hand-held knives such as the Goulian or Weck knife [2]. Adequate debridement with these knives is determined by the presence of punctuate bleeding and a viable dermis. This procedure is not only associated with substantial blood loss, but also with the unnecessary removal of viable dermis [2, 3]. Loss of dermis has been considered one of the main factors determining the quality of the scar and the degree of contraction of the healing wound [4–6]. Therefore, methods which maximally preserve dermis are essential. During the last decade, hydrosurgery has become popular in burn surgery as a new option for excision of non-viable tissue prior to skin grafting [7–9]. The Versajet™ hydrosurgery system (Smith and Nephew, St. Petersburg, FL, USA) was developed in 1997 for the purpose of debriding various types of wounds, including burn wounds, and is superseded by the Versajet II™ (Smith and Nephew) in 2011 [8]. The Versajet II™ system works by producing a high-pressure jet of water across an aperture in an angled handpiece. The Venturi effect creates a vacuum that removes surface debris which is sucked into the machine together with the irrigation fluid. The cutting and aspiration effects can be controlled by adjusting console power settings, handpiece orientation, and handpiece pressure. The vacuum that is created by the speed of the jet aims to lift only non-viable tissue and thus maximal dermal preservation could be achieved. For this reason, hydrosurgical debridement of burns might lead to a better scar outcome compared to conventional sharp debridement.

Although burn specialists widely use hydosurgery as an alternative for conventional tangential debridement [6, 7] only a limited number of studies are available on the effects of hydrosurgery in burn patients [10–12]. A guideline from the National Institute for Health and Care Excellence (NICE) recently reported that the Versajet™ is an efficient and safe wound debridement tool in both adults and children with acute and chronic wounds [8]. Up to now, two randomized controlled trials comparing hydrosurgical and conventional debridement in patients with burns have been published [13, 14].

Gravante et al. stated that adequate debridement of the wound bed was possible in all patients treated with the Versajet™ system [13]. The authors suggested that hydrosurgical excision was more precise in obtaining the correct dermal plane, but did not confirm this with objective measurements. Hyland et al. studied children under the age of 16 years and histologically confirmed that significantly more viable dermis was preserved in the group of patients treated with hydrosurgery compared to the conventionally treated group of patients [14]. However, they did not observe significant differences in scar quality measured with the Vancouver Scar Scale (VSS) at 3 and 6 months post burn. Furthermore, they did not use any objective scar measurement tools and the study was limited by a relatively short follow-up period as scars mature over a period of at least 1 year [15, 16]. Also, the VSS was formally not designed to indicate burn scar severity, has a moderate reliability and does not include the opinion of the patient [17]. Hence, it remains unclear whether hydrosurgery for the routine debridement of deep dermal burns prior to skin grafting leads to increased dermal preservation and better scar quality outcomes.

The aim of this study, therefore, is to assess the effectiveness of hydrosurgical compared to conventional debridement in deep dermal burns. Long-term scar quality after hydrosurgical and conventional debridement of deep dermal burns in relation to histologically measured dermal preservation will be analyzed.

## Methods/design

### Protocol and registration

**Fig. 1:**
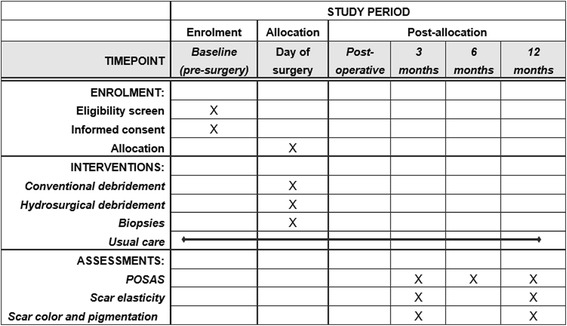
Schedule of enrollments, interventions and assessments

The study was approved by the Medical Ethics Committee (NL58875.101.16) and by the Institutional Review Boards of each participating burn center. The methods applied were specified in advance, documented in a protocol, and registered (http://www.trialregister.nl, NTR6232). The protocol has been designed in accordance with the SPIRIT (Standard Protocol Items: Recommendations for Interventional Trials) guidelines for interventional trials [18]. The SPIRIT Checklist and Figure are given in Additional file 1 and Fig. 1, respectively.

### Study design

A multicenter, randomized controlled trial with an intra-patient comparison of hydrosurgical and conventional debridement will be conducted in the three Dutch burn centers: Rotterdam, Beverwijk, and Groningen. As the healing process of burn wounds and scar formation differs between patients we chose an intra-patient design to provide representative outcomes and to limit inter-patient bias.

### Participants

Patients of all ages with deep dermal burns and an indication for tangential excision and skin grafting are eligible for this trial, either hospitalized or under treatment in the outpatient clinic of one of the participating burns centers. Exclusion criteria are as follows: a burn wound < 50 cm^2^, total body surface area (TBSA) burned > 30%, full-thickness burns, chemical or electrical burns, infected wounds (clinical symptoms in combination with positive wound swabs), patients with insufficient knowledge of the Dutch or English language, patients who are unlikely to comply with requirement of the study protocol and follow-up, and patients who are (temporarily) incompetent because of sedation and/or intubation.

Patients are included after receiving a full, understandable and neutral explanation of the study by a member of the research team and after having given written informed consent.

### Interventions

#### Conventional tangential excision

Tangential excision with guarded knives relies on the stepwise excision of a layer of tissue using a flat blade. The addition of a guard prevents the removal of excessive amounts of tissue, and most of these knives allow adjustment of the width of the gap between the blade and the guard. However, if the gap is too narrow the instrument will glide off the burn without any debridement taking place [2].

#### Hydrosurgical tangential excision

The Versajet™ II hydrosurgery system (Smith and Nephew. St. Petersburg, FL, USA) was CE marked in 2011 and was launched in 2012 [8]. It uses a high-pressure jet of sterile saline to debride wounds. It is attached to a console, which is then operated by a foot pedal. The saline is forced out of a narrow nozzle and functions like a knife which allows debridement and aspiration of debris to occur simultaneously. Pressure can be adjusted (power setting 1–10) to facilitate the desired depth of debridement. As a result, the correct level might be reached more accurately, preserving as much dermis as possible. Hydrosurgery is preferentially suited to debride softer necrotic tissues, and cannot be used to debride full-thickness burns as it does not cut through hard eschar. Versajet™ is reported to be used routinely in multiple centers around the world these days [8]. Nevertheless, a clear algorithm for its use is lacking, and burn specialists may choose individually whether hydrosurgery can be applied or not [9].

### Surgical procedure

**Fig. 2:**
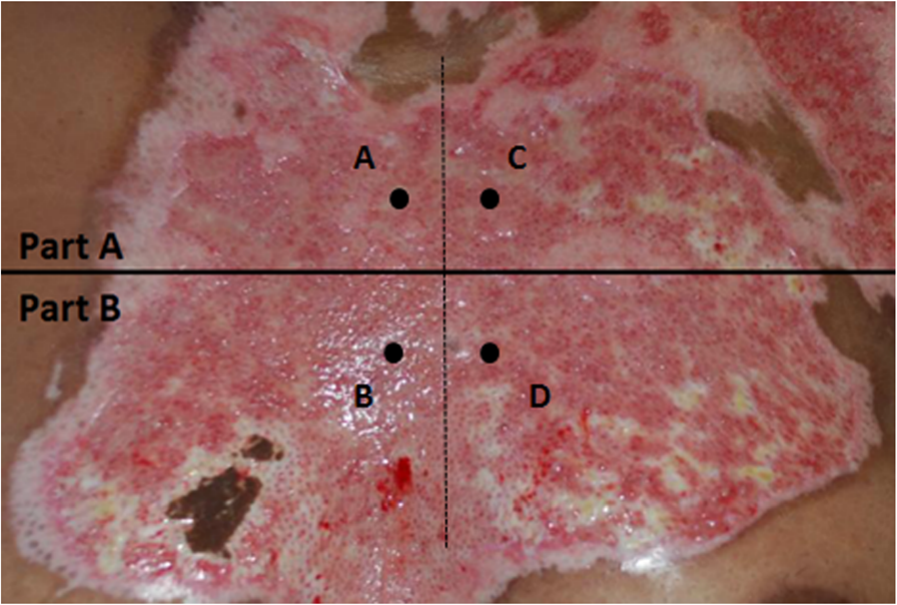
Location of punch biopsies. **a** Biopsy part A, before debridement. **b** Biopsy part B, before debridement. **c** Biopsy part A, after debridement. **d** Biopsy part B, after debridement

Prior to surgery, the surgeon divides the study area into two adjacent parts of equal size and burn depth (part A and part B). These parts are randomly allocated to either conventional or hydrosurgical (Versajet**™**) tangential debridement. Two 3-mm punch biopsies of both intervention areas will be collected before and after debridement, according to a standardized method (Fig. 2), to determine the amount of viable dermis before and after excision. Type of mesh graft and expansion will also be standardized per patient to ensure an equal mesh cover of the two intervention areas. Before and after surgery, standard wound care is given. After discharge, patients will be treated in an outpatient setting according to the local protocol.

### Study outcomes

#### Primary outcome measure

Our primary outcome measure is scar quality assessed by the items of the observer scale of the Patient and Observer Scar Assessment Scale (POSAS) at 12 months post surgery. The POSAS is recognized as a highly reliable scar rating scale, and consists of two numeric scales: the Patient Scar Assessment Scale (patient scale) and the Observer Scar Assessment Scale (observer scale) [19, 20]. The observer scale contains the items vascularization, pliability, pigmentation, thickness, and relief. All items will be measured for part A and part B of the study wound on a 10-point rating scale by two experienced, trained, and independent observers to improve the reliability of the assessment. The average of the observers’ scores will range from 1, which corresponds to the situation of normal skin, to 10, indicating the “worst” imaginable scar.

#### Secondary outcome measures

##### Subjective scar assessment

Scar quality of part A and part B of the study area will be measured at 3, 6, and 12 months post surgery using the POSAS. Although the total score of the observer scale at 12 months is our primary outcome, the items of the patient scale of the POSAS will also be measured and analyzed separately. The patient scale of the POSAS contains the items color, pliability, thickness, relief, itching, and pain. These items will be scored on a 10-point rating scale and added to form the total patient score. In addition, patients and observers will score their overall opinion on the scar (1–10, numeric scale), and total scores of the patient and observers will be added to form a total score.

##### Scar elasticity

Scar elasticity will be measured with the Cutometer® (Courage-Khazaka electronic GmbH Cologne, Germany). The Cutometer® is a validated instrument to measures the viscoelasticity of the skin by analyzing its maximal extension (Uf in millimeters) in response to negative pressure [21].

##### Scar color and pigmentation

Scar color and pigmentation will be measured with the Dermaspectrometer® (Cortex Technology ApS Hadsund, Denmark), which is a reliable narrowband spectrometer that computes an erythema and melanin index [19].

Measurements with the Cutometer® and Dermaspectrometer® are performed at 3 and 12 months post burn on both parts (A and B) of the study area, and adjacent normal skin. For objective data collection, measurements will be performed following a fixed protocol.

##### Dermal preservation

During surgery, two punch biopsies will be taken out of both parts (A and B) of the study area, pre and post debridement, using a 3-mm punch. The biopsies will be fixed in kryofix and processed into 3–5-μm histological slides. Sections will then be stained with hematoxylin and eosin (H&E) or picrosirius red. To determine the amount of dermal preservation and hence precision of debridement, the amount of viable tissue on pre- and post-debridement specimens will be recorded using light microscopy.

##### Baseline characteristics

Data registration will start on the day of randomization. Data regarding patients’ baseline characteristics will be obtained from patients’ medical records:

- Demographics: age, gender
- Burn characteristics: percent TBSA burned, affected anatomical site(s), wound etiology, time to surgery, and burn depth of the study area. If possible, burn depth will accurately be determined on days 2–5 post burn by clinical evaluation and laser Doppler imaging (LDI) scan using the moorLDI2-Burn Imager™ (Moor Instruments, Axminster, UK) or similar [22]
- Clinical characteristics: skin type according to the Fitzpatrick skin type scale; wound healing time (measured in days till 95% re-epithelization); comorbidity; Weck knife, Versajet™, and dermatome settings; expansion of skin graft; adverse events (graft loss, wound infection); and need for reconstructive surgery.

### Sample size

Power calculation is based on the results obtained by an unpublished retrospective study on scar quality after hydrosurgery versus guarded knife excision in the Martini Hospital in Groningen [9]. The primary outcome measure was scar quality assessed from photographs, and expressed in the total score of the observer part of the POSAS. Because scar quality was assessed from photographs, pliability was not taken into account [23]. Therefore, scar assessment contained the four items vascularization, pigmentation, thickness, and relief.

The lowest sum score, reflecting normal skin, was 4 and the highest score, reflecting the worst imaginable scar, was 40. In this study, the observer score of the POSAS questionnaire 12 months post surgery was 14.7 in the hydrosurgery groups versus 16.7, with a pooled standard deviation (SD) of 6.53. This results in an effect size of 0.3. Because of the within-subject design, a correction for correlated samples was included, assuming a correlation of 0.4 between POSAS observer score within one patient. Given a power of 0.8 and a level of significance of 0.05 a number of 105 patients is needed. Assuming a 30% dropout, 137 patients need to be recruited.

### Randomization

Randomization will occur in the operating theater after the wound is divided into two comparable study areas, defined as part A and part B. These areas are randomly assigned to receive either hydrosurgical or conventional debridement. Allocation of the treatment will be stratified per institute in blocks using the online randomization program CASTOR, https://data.castoredc.com. The outcome will be displayed on the website, only visible for the person who performed the randomization and the principal investigator. After randomization, the central trial coordinator will receive instructions with the inclusion number.

### Blinding

Patients are blinded as they are sedated during surgery and will not be aware which treatment they received on which part of the wound. Blinding surgical treatment is not possible, as the burn surgeon knows which part of the wound received which surgical treatment. Outcome assessment is blinded as the member of the research team who performs the follow-up measurements is unaware of the technique used for debridement of part A or part B. In case of randomization-related difficulties, the central trial coordinator can be contacted.

### Statistical analysis

Data analysis will be performed using SPSS PASW Statistics 23.0 (IBM, New York City, NY, USA).

#### Primary outcome

Differences in scar quality 12 months post surgery, assessed as the total score of the observer scale of the POSAS, will be analyzed using the paired Student *t* test (in case of normal distribution) or the Wilcoxon signed-rank test (non-normal distribution).

#### Secondary outcomes

Differences in 12-month outcomes of the patient scar assessment, the observer scar assessment, scar elasticity (measured by the Cutometer®), scar color (measured by the DermaSpectrometer®) and differences in viable dermis after excision (measured by histopathology) will be analyzed using the Wilcoxon signed-rank test in case of non-normal distribution, or the paired Student *t* test in case of normal distribution. Because of repeated measurements within patients and loss to follow-up, overall differences of scar quality measurements will be analyzed using generalized estimating equations, with unstructured working correlation matrix structure.

## Discussion

In this paper, we have described the design of our study into long-term scar quality after hydrosurgical and conventional tangential excision of deep dermal burns. This will be the first study that assesses differences in scar quality between both treatment modalities at 12 months post surgery in both adults and children with subjective and objective measurement tools.

Subjective scar quality will be assessed using the POSAS. The POSAS is unique as it takes the opinion of the patients into account which is mandatory for a clinically relevant scar evaluation [24].

Scar quality will not only be measured subjectively, but also with objective measurement tools concerning scar pigmentation, vascularity, and pliability. Aside from these evaluations, we want to support our results via quantitative analysis of the histological specimens. For a reliable follow-up, documentation of which area of the wound is part A, and which area is part B needs to be specific to allow accurate assessment of the correct areas, as it is possible that there might be no differences visible at follow-up.

In this study, an accurate diagnosis of wound depth is essential to determine the indication for surgery. Therefore, all three burn centers are in possession of an LDI scan to assess burn wound depth, which has an accuracy of 95% in combination with a clinical evaluation of the wound [22, 25, 26]. Moreover, it can be used to make sure that part A and part B of the study area are of equal burn depth. To enhance the applicability and generalizability of this trial, we chose a multicenter trial design and will recruit patients treated in one of the three national Dutch burn centers. To increase generalizability, and because of the intra-patient design, we are forbearing regarding local clinical care; for example, timing of surgery and type of wound dressings. This study will contribute to the optimal surgical treatment of patients with deep dermal burn wounds and the results should be of high international value, as hydrosurgery is used worldwide.

### Trial status

This manuscript is a restructured and edited version of the REC approved protocol (version 3.2, 6 February 2017) to comply with the SPIRIT guidelines. Recruitment opened on January 10, 2017, and is expected to be completed in January 2019. As of 16 April 2018, 56 patients had been recruited.

## Additional file

Additional file 1: SPIRIT Checklist. (DOC 120 kb)

## Abbreviations

LDI: Laser Doppler imaging
NICE: National Institute for Health and Care Excellence
POSAS: Patient and Observer Scar Assessment Scale
REC: Research Ethics Committee
SPIRIT: Standard Protocol Items: Recommendations for Interventional Trials
TBSA: Total body surface area
VSS: Vancouver Scar Scale
